# Handheld ultrasound-guided 20 mm basket Intact breast lesion excision system biopsy for excision of benign breast lesions

**DOI:** 10.1186/bcr3789

**Published:** 2015-11-05

**Authors:** Simon Lowes, Alice Leaver, Alice Townend, Jackie Westgarth, Peter Newton, Dianne Hemming, Alan Redman

**Affiliations:** 1 Gateshead Hospitals NHS Trust, Gateshead, UK

## Introduction

In selected patients, our Unit has recently moved from handheld ultrasound-guided vacuum-assisted core biopsy (VACB) piecemeal acquisition of tissue to the handheld Intact Breast Lesion Excision System (Intact). Intact removes a single piece of tissue, potentially allowing radiologists to excise the entire lesion as well as allowing pathologists to visualise lesion architecture more easily and to calculate margins. We evaluated our early experience of excising benign or likely benign breast lesions using the 20 mm Intact.

## Methods

Prospective data collection was performed on all patients undergoing handheld ultrasound-guided Intact excision under local anaesthetic in 2014 and 2015, which comprised 19 lesions in 18 female patients aged 29−73.

## Results

The device was technically straightforward to operate and well-tolerated by patients with no significant complications. Handheld needle orientation was difficult within dense glandular tissue (only one acquisition is possible per needle), but improved with increased operator experience. Achieving adequate analgesia required higher quantities of local anaesthetic than for the equivalent VACB. Pathologists found specimens easier to interpret than VACB samples. In all cases adequate excision was completed sonographically at one outpatient appointment, but in six cases a second Intact biopsy and/or a VACB was required to complete that excision, with extra cost implications. In two patients with M3 microcalcification the Intact pathology demonstrated ductal carcinoma in situ, leading to surgical wide local excision.

## Conclusion

Our early experience shows Intact as a reliable and effective tool for handheld diagnostic and/or therapeutic excision of selected breast lesions.

